# In vitro study of the PLA2 inhibition and antioxidant activities of *Aloe vera *leaf skin extracts

**DOI:** 10.1186/1476-511X-10-30

**Published:** 2011-02-11

**Authors:** Maher Kammoun, Sonia Miladi, Yassine Ben Ali, Mohamed Damak, Youssef Gargouri, Sofiane Bezzine

**Affiliations:** 1 Laboratoire de Biochimie et de Génie Enzymatique des Lipases, Ecole Nationale d'Ingénieurs de Sfax, University of Sfax, Tunisia; 2 Laboratoire de Chimie des Substances Naturelles, Faculté des Sciences de Sfax, University of Sfax, Tunisia

## Abstract

**Background:**

In the present work we determined the total phenolic content of *Aloe vera *leaf skin (AVLS) extracts by using various solvents (hexane, chloroform-ethanol (1/1), ethyl acetate, butanol and water). We have also evaluated the antioxidant and the anti-PLA2 properties of these extracts by measuring their inhibition potency on the human pro-inflammatory phospholipase A2 (group IIA).

**Results:**

The water extract exhibits the highest inhibitory effect with an IC_50 _= 0.22 mg/ml and interestingly no effect was observed on the digestive phospholipase A2 (group IB) even at a concentration of 5 mg/ml. Antioxidant activities were also analyzed and the most active extracts were observed when using chloroform ethanol (1/1) and ethyl acetate (IC_50 _= 0.274 and 0.326 mg/ml, respectively). Analysis of the total phenolic content reveals that the water extract, with the best anti-PLA2 effect, was poor in phenolic molecules (2 mg GAE/g). This latter value has to be compared with the chloroform-ethanol and the ethyl acetate extracts (40 and 23.8 mg GAE/g, respectively), mostly responsible for the antioxidant activity.

**Conclusion:**

A significant correlation was established between the total phenolic content and the antioxidant capacity but not with the anti PLA2 activity. Results from phytochemical screening suggest that the anti PLA2 molecules were probably catechin tannins compounds.

## Background

*Aloe vera *L. (syn.: *Aloe barbadensis *Miller) is a perennial succulent plant belonging to the Aloeaceae family (sub-family of the Asphodelaceae) [1]. Among over 400 *Aloe *species, *Aloe vera *is the most accepted specie for various medical, cosmetic and neutraceutical purposes [2-4]. The plant is made of turgid green leaves joined at the stem in a rosette pattern. Each leaf consists of two parts: an outer green rind (skin) and an inner clear pulp (gel). The plant was described to contain a large amount of phenolic compounds [2,5-10] with a high content of 1,8-dihydroxyanthraquinone derivatives (aloe emodin) and glycosides (aloins), which were used as cathartic [11-13]. Various studies have revealed that *Aloe vera *leaf skin *(AVLS*) possesses many pharmaceutical properties, including purgative [14], antibacterial [15,16], anticancer [17-19], antifungal [20] and antioxidant [21-25].

The secreted phospholipases A2 (sPLA2) are low molecular mass enzymes (14-19 kDa) with a rigid tertiary structure due to five to eight disulfide bonds that probably confer resistance against proteolysis and thermal denaturation [26,27]. These enzymes hydrolyze the *Sn-2 *ester bond of glycerophospholipids to liberate free fatty acid and lysophospholipid. So far, eleven sPLA2 have been described in mammals and they belong to groups IB, IIA, IIC, IID, IIE, IIF, III, V, X, XIIA and XIIB. The first non pancreatic mammalian identified sPLA2 was the group IIA enzyme which plays important roles in the initiation and amplification of the inflammatory reaction. Arachidonic acid is metabolized by either the cyclo-oxygenase or the lipo-oxygenase enzymatic pathway to produce diverse families of eicosanoids including prostaglandins and leukotrienes are involved in inflammation related processes [28,29].

Based on the central role of lipid mediators in the inflammatory processes, the potential value of controlling phospholipid metabolism through PLA2 inhibition has always been acknowledged [30]. Numerous compounds have been proposed as inhibitors of various sPLA2. However, clinical studies have never reached a therapeutical stage. The aim of this study was firstly to investigate the phytochemical composition of AVLS and secondly to evaluate some biochemical activities of the ethanolic extracts of AVLS such as their antioxidant capacity and their inhibitory effects on the pro-inflammatory phospholipase A2 group IIA. As a control experiment, we used the digestive pancreatic phospholipase A2 group IB.

## Results

### Extraction yields and phytochemical screening of Aloe vera leaf skin

*Aloe vera *leaf skin was harvested in august and the extraction yields were summarized in table 1. These results show the various phytochemical families present in AVLS fractions. Tests for steroids, terpenoids and carotenoids were positive in both hexane and ethyl acetate fractions. Anthraquinones were mostly detected in the ethyl acetate, chloroform-ethanol and butanol fractions while alkaloids and flavonoids were absent in all fractions. It is worth noticing that catechin tannins were only detected in the aqueous extract.

**Table 1:** Phytochemical screening and yields of *AVLS *extracts.

Extracts	Hexane	Ethyl acetate	Chloroform-ethanol	Butanol	Water
**Yields (%)**	**5.10**	**11.20**	**6.09**	**8.57**	**66.67**

**Steroids**	++	+	-	-	-
**Terpenoids**	++	++	-	-	-
**Carotenoids**	+	+	-	-	-
**Anthraquinones**	-	++	+++	+	-
**Flavonoids**	-	-	-	-	-
**Alkaloids**	-	-	-	-	-
**Catechin tannins**	-	-	-	-	+++

The yields were determined as relative to the initial amount of dry ethanolic extract (79.8 g).

-: Absent, **+**: Present, **++**: Rich, **+++**: Very rich

### Total phenolic contents

Results of the total phenolic contents from the various extracts, expressed as milligram of GAEs per gram of extract, were presented in table 2. Among the five extraction systems used, the chloroform-ethanol extract showed the highest amount of phenolic compounds (40.5 mg GAE/g) and the poorest one was the water extract which contained only 2.07 mg GAE/g.

**Table 2:** Total phenol content and IC_50 _on DPPH of *AVLS *extracts

Extracts	TPC (mg GAE/g of extract)	**IC**_**50 **_**on DPPH radical (mg/ml)**
**Hexane**	9.600 ± 0.014	0.366
**Ethyl acetate**	23.800 ± 0.058	0.326
**Chloroform-ethanol (1/1)**	40.500 ± 0.041	0.274
**Butanol**	16.900 ± 0.039	0.635
**Water**	2.072 ± 0.002	>1

**BHT**	-	69 10^-3^
**α-tocopherol**	-	7.5 10^-3^

IC_50 _values on DPPH were calculated from the plot of the scavenging effect against the extract concentration. Effects of BHT and α-tocopherol, used as standard, were measured in the same conditions. All experiments were performed in triplicate ± standard deviation

### Antioxidant activity

#### DPPH radical scavenging activity

The antiradical activities of extracts were determined using the DPPH free radical assay. After the measurement of absorbance at 517 nm, the radical scavenging activities of the various extracts were expressed as the mean of the IC_50 _values (mg/ml). IC_50 _values of the five *AVLS *extracts, α-tocopherol and BHT are reported in table 2.

Our results show that the chloroform-ethanol (1/1) extract of leaf skin exhibits the highest capacity to reduce DPPH (IC_50 _= 0.274 mg/ml), followed by the ethyl acetate extract (IC_50 _= 0.326 mg/ml) and the hexane extract (IC_50 _= 0.366 mg/ml). The lowest antiradical capacity was found in the water extract. For the sake of comparison, we measured the IC_50 _value of BHT and α-tocopherol which were 69 and 7.5 μg/ml respectively.

#### Reducing power assay

**Figure 1:**
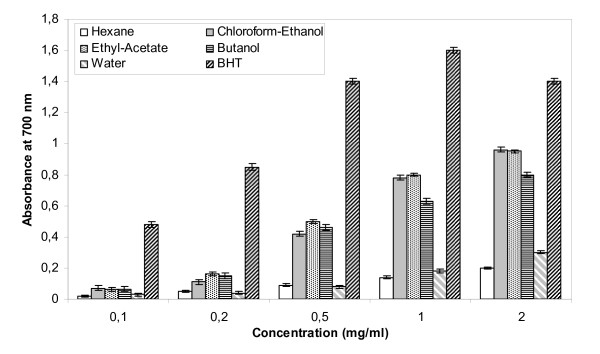
**Reducing power of *AVLS *measured with hexane, chloroform-ethanol, ethyl acetate, butanol and water extracts as compared to BHT**. Experiments were performed in triplicate ± standard deviation.

Results presented in Figure 1 show the reducing power of the *AVLS *fractions and BHT, used as reference. As expected, the reducing power of all samples tested is proportional to their concentrations. Below 0.2 mg/ml no significant difference was seen among all extracts. However differences were more pronounced with extracts at concentrations higher than 0.5 mg/ml. According to the results presented in Figure 1, ethyl acetate, chloroform-ethanol and butanol extracts were found to be better radical reducer (with electron donating capacities) as compared to water and hexane extracts. However, these extracts are less effective than BHT, a widely used commercial antioxidant which exhibits a high reducing capacity even when used at very low concentrations (0.05 mg/ml).

### Evaluation of PLA2 inhibitory effect

To evaluate the potential anti-inflammatory activity of AVLS, we tested the inhibitory effects of various extracts using two secreted phospholipases: hG-IIA involved in the inflammatory process and the pG-IB which hydrolyzes dietary phospholipids. Our main objective was to find an extract which was able to inhibit selectively the pro-inflammatory phospholipase A2 group IIA with no or minimal inhibitory effect on the digestive phospholipase A2 group IB.

**Figure 2:**
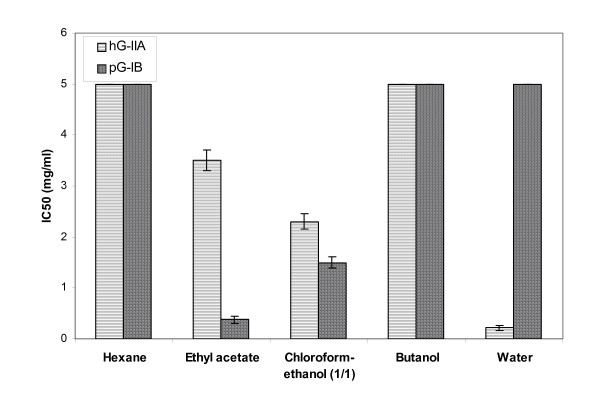
**IC**_**50 **_**of AVLS extracts measured during the inhibition of hG-IIA and pG-IB**. Experiments were performed in triplicate ± standard deviation.

Out of the 5 extracts screened, three of them (ethyl acetate, chloroform-ethanol (1/1) and water) showed significant results (Figure 2). It is worth noticing that the water extract shows the most promising results in inhibiting the catalytic activity of the hG-IIA with an IC_50 _of 0.22 mg/ml. In sharp contrast, using the same extract even at concentrations higher than 5 mg/ml, no inhibition of the phospholipase A2 activity of pG-IB was noticed. These results indicate a selective inhibition of the water extract against these two sPLA2.

### Correlation significance study

It was worth studying the potential correlations between the phenolic content of AVLS extracts with their antioxidant capacity and their inhibition of phospholipase A2 activity, since it was previously reported that phenolic compounds contribute directly to the antioxidant activity [31] and to the anti-inflammatory effects [32].

A correlation analysis was performed between the total phenolic content, the antioxidant activity and the phospholipase A2 inhibition described in the present study. Results reported in table 3 show a linear regression and a significant relationship between the total phenolic content and free radical scavenging or reduction power (r = 0.9, P < 0.05). As expected, our results indicate that in the presence of high concentrations of the phenolic compounds, the antioxidant activity increases significantly. Furthermore, a positive correlation was also noticed between the total phenolic content and their inhibitory effect on pG-IB (r = 0.78, P < 0.1) but no significant correlation was obtained between the phenolic content and the hG-IIA inhibition.

**Table 3:** Correlation significance between the total phenolic content, antioxidant capacity and phospholipase A2 inhibition

Parameter	Reducing power	**IC**_**50 **_**value of scavenging activity**	**IC**_**50 **_**value of hG-IIA inhibition**	**IC**_**50 **_**value of pG-IB inhibition**
**Number of XY Pairs**	5	5	5	5
**Spearman r**	0,9	-0,9	0,0513	-0,7826
**P value (one-tailed)**	0,0417	0,0417	0,475	0,0667
**P value summary**	*	*	ns	*
**Exact or approximate P value?**	Exact	Exact	Exact	Exact
**Is the correlation significant? (alpha = 0.1)**	Yes	Yes	No	Yes

## Discussion

The heterogeneity of the phytochemical composition of AVLS extracts is very promising for future medical applications. In fact, the chloroform-ethanol extract, which is rich in phenolic compounds (40.5 mg GAE/g), was the best antioxidant tested. In contrast, the water extract which is the poorest in phenolic compounds (2.07 mg GAE/g) did not show any antioxidant activity. This finding suggests that phenolic compounds from AVLS are responsible for the antioxidant effect. These results in agreement with those obtained by Kahkonen et al. (1999) [33], Shahidi and Marian (2003) [34] who reported that the differences in antioxidant activities of plant extracts could be due to the variable contents of their phenolic compounds. In the search for natural anti-inflammatory compounds, several studies were performed using *Aloe vera *due to its well known potent anti-inflammatory effects. Several authors have demonstrated the anti-inflammatory effect of this plant. Habeeb et al (2007) [35] have shown that the inner leaf gel from *Aloe vera*, when using an extract at a concentration of around 45 mg/ml, can suppress the cytokine induced inflammation after a whole bacterial stimulation of the human immune cells.

We also report in this study that the water extract of AVLS possesses the best inhibitory effect on the pro-inflammatory PLA2 group IIA with an IC_50 _= 0.22 mg/ml. The phytochemical analysis showed that this water extract was very rich in catechin tannins suggesting that, apart from its low concentration in polyphenol contents, the molecules responsible for the anti-PLA2 effects belong probably to different chemical families. Probably the catechin tannins, rich in the water extracts and responsible for the anti-PLA2 activities, are different from the molecules bearing the antioxidant effect. It was previously reported that LY311727 inhibit hG-IIA and pG-IB with an IC50 of 0.47 and 8 μM, respectively [36]. In the same case, Me-Indoxam was found to be the most potent inhibitor of sPLA2 and was extensively studied. It is able to inhibit hG-IIA with an IC50 of 0.006 μM and its IC50 on hG-IB is about 6 μM [37].

Several studies evaluated the relationships between the antioxidant activity of plant extracts and their phenolic content. Velioglu et al. (1998) [38] reported a significant relationship between the total phenolic content and its antioxidant activity in selected fruits, vegetables and grain products.

In agreement with these latter results, we report here a significant correlation between the total phenolic compounds and their antioxidant effects (r = 0.9, P < 0.05) as well as during pG-IB inhibition (r = 0.78, P < 0.1). In contrast, no correlation was observed between the phenolic content and the IC_50 _measured during hG-IIA inhibition. These results suggest that different compounds are responsible for the inhibition of these two groups of sPLA2. In addition, the catechin tannins, present in the water extract, could be responsible for the inhibition of the hG-IIA activity and not that of pG-IB. Several authors have suggested that compounds responsible for the anti-inflammatory effect could not be phenolic molecules. Yagi et al (2003) [39] reported that neutral polysaccharides, aloemannan and acemannan isolated from *Aloe vera*, exhibited antitumor, anti-inflammatory and immunosuppressive activities. Similar results were obtained by Parc et al., (2009) [40] who demonstrated that Aloe-emodin, an anthraquinone compound, extracted from *Aloe vera *has an anti-inflammatory activity by blocking inducible nitric oxide synthase and cyclooxygenase-2 mRNA expression. Moreover, Esua et al (2005) [41] have isolated two maloyl glucans with significant effect against the gene expression of very important pro-inflammatory cytokines as well as counteracting the effect of the tumor necrosis factor-α.

## Materials and methods

### Enzymes and reagents

Human group IIA phospholipase A2 (hG-IIA) and Me-Indoxam were generous gifts from Dr. Gerard Lambeau (IPMC, France). Porcine group IB phospholipase A2 (pG-IB), Egg yolk phosphatidylcholine or lecithin, red phenol and sodium taurodeoxycholate (NaTDC), 2,2-Diphenyl-1-picrylhydrasyl (DPPH), potassium phosphate, BHT (butylated hydroxytoluene), α-tocopherol and gallic acid were purchased from Sigma Chemical Co. (St.Loui, MO). Potassium ferricyanide, ferric chloride and Folin-Ciocalteu phenol reagent were purchased from Merck. Visible spectra measurements were performed using Anadéo visible spectrophotometer (Anadéo-Bibby).

### Extraction and phytochemical screening

Mature fresh leaves of *Aloe vera *(Aloaceae) with an approximate length of 0.5 to 0.7 m were collected from the region of Kairouan, (Tunisia) and harvested in August 2008. The fresh *Aloe vera *leaf skin (3 kg) was washed with distilled water and was extracted with 5 liters of ethanol (95%) by maceration for 48 hours at room temperature. After a filtration step, the ethanolic extract was concentrated under reduced pressure and lyophilized to yield the ethanolic extract (79.80 g). This extract was suspended in water (200 ml) and partitioned successively with hexane (1 liter), ethyl acetate (4 liters), chloroform-ethanol (1/1, v/v) (1.5 liters) and butanol (1.5 liters). The remaining solution is designated "water extract".

Phytochemical screening was carried out to identify the chemical compounds present in the AVLS extract, in order to determine the chemical nature of the potential active principles responsible for the observed biochemical effects [42,43].

### Inhibition of sPLA2 activity

The test of inhibitory activity of PLA2 was performed as described by De Aranjo and Radvany (1987) [44]. Briefly, the substrate consisted of 3.5 mM lecithin in a mixture of 3 mM NaTDC, 100 mM NaCl, 10 mM CaCl_2 _and 0.055 mM red phenol as colorimetric indicator in 100 ml H_2_O. The pH of the reaction mixture was adjusted to 7.6. The hG-IIA or the pG-IB were solubilized in 10% acetonitrile at a concentration of 0.02 and 0.002 μg/μl; respectively. A volume of 10 μl of these PLA2 solutions was incubated with 10 μl of each AVLS extract for 20 min at room temperature. Then, 1 ml of the PLA2 substrate was added, and the kinetic of hydrolysis was followed during 5 min by reading the OD at 558 nm. The inhibition percentage was calculated by comparison with a control experiment (absence of AVLS extract) and the IC_50 _values were determined from the curve.

### Total phenols determination

The total phenolic content (TPC) of the fractions of *Aloe vera *leaf skin (*AVLS*) was estimated by a colorimetric assay, according to the method described by Singleton & Rossi (1965) with some modifications [45]. Briefly, 1 ml of sample at 1 mg/ml was mixed with 1 ml of Folin-Ciocalteu reagent. After 3 min of incubation, 1 ml of saturated Na_2_CO_3 _solution was added and the volume was adjusted to 10 ml with distilled water. The reaction mixture was kept in the dark for 90 min, after which the absorbance was read at 725 nm. The TPC was determined using gallic acid as a standard.

### Antioxidant testing assays

The antioxidant activity of *AVLS *fractions was determined by using the following methods:

#### DPPH radical scavenging assay

The antioxidant activity of the *AVLS *fractions was measured as equivalent of hydrogen-donating or radical scavenging ability, using the DPPH method [46-48] with some modifications. Briefly, 1.5 ml of DPPH solution at 10^-5 ^M was incubated with 1.5 ml of *AVLS *extracts containing variable amounts of dry weight (between 0.01 and 1 mg). The reaction mixture was shaken and incubated in the dark for 30 min at room temperature. The control experiment was performed as described above without adding any AVLS extract. The absorbance of the solution was measured at 517 nm. The radical scavenging activity was calculated using the following equation:

$$\text{Scavenging effect }\left(\%\right)=(1-\frac{{\text{A}}_{\text{Sample}}}{{\text{A}}_{\text{Control}}})\times \text{1}00$$

The extract concentration providing 50% inhibition (IC_50_) was calculated from the plot of the scavenging effect (percentage) against the extract concentration. BHT and α-tocopherol were used as standards.

#### Reducing power assay

The reducing power of *AVLS *fractions was determined according to the method of Oyaizu (1986) [49]. Solutions of variable concentration of *AVLS *extracts were mixed with 1 ml of 0.2 M sodium phosphate buffer at pH 6.6 and 1 ml of 1% potassium ferricyanide (K_3_Fe(CN)_6_). The obtained reaction mixture was then incubated at 50°C for 20 min. Next, 1 ml of 10% (w/v) trichloroacetic acid was added to the mixture which centrifuged at 3000 rpm for 10 min. The upper layer solution (2.5 ml) was mixed with 2.5 ml of deionised water and 0.5 ml of fresh ferric chloride at 0.1%. The absorbance was measured at 700 nm. Higher absorbance of the reaction mixture indicates greater reducing power.

### Statistical analysis and correlation study

Experimental results were given as mean value ± SD of three separate experiments. Statistical analysis was conducted using Microsoft Excel software. Differences at *P *< 0.05, using student's *t*-test, were considered to be significant.

Correlation study was performed using the Graphic Pad Prism software, version 5.01.

## Abbreviations

TPC: total phenol content; AVLS: Aloe vera leaf skin; DPPH: 2,2-diphenyl-1-picrylhydrazyl; NaTDC: sodium taurodeoxycholate; BHT: Butylhydroxytoluene; GAE: Gallic acid equivalent; PLA2: Phospholipase A2

## Competing interests

The authors declare that they have no competing interests.

## Authors' contributions

MK and SM carried out all the studies, analyzed the data and drafted the manuscript. YBA helped with the analysis of the data and to correct the manuscript. YG helped with the discussion of the data and the correction of the manuscript. MD helped with the discussion. SB participated in the study design and helped to draft the manuscript. All authors have read and approved the final manuscript.
